# Multidrug-Resistant Bloodstream Infections Before and After COVID-19: A Temporal Assessment of Outcomes and Resistance Across Two Eras in a Non-COVID Intensive Care Unit—The MATURATE ICU Study

**DOI:** 10.3390/microorganisms14092098

**Published:** 2026-09-19

**Authors:** Sotiria Kefala, Foteini Fligou, Ioannis Chandroulis, Marina Amerali, Stamatia Tsoupra, Eirini Zarkadi, Eleni Polyzou, Panagiota Pnevmatikou, Fevronia Kolonitsiou, Karolina Akinosoglou

**Affiliations:** 1 Department of Anesthesiology and Intensive Care, University General Hospital of Patras, 26504 Rio, Greece; sotiria_kefala@yahoo.gr (S.K.); fflig@upatras.gr (F.F.); amerali.marina@gmail.com (M.A.); stamtsoupra@gmail.com (S.T.); edzarka@gmail.com (E.Z.); 2 Faculty of Medicine, University of Patras, 26504 Rio, Greece; kolonits@upatras.gr (F.K.); akin@upatras.gr (K.A.); 3 School of Science and Technology, Hellenic Open University, 26335 Patras, Greece; std528050@ac.eap.gr; 4 Department of Internal Medicine, University General Hospital of Patras, 26504 Rio, Greece; 5 Department of Microbiology, University General Hospital of Patras, 26504 Rio, Greece; pnevmatikoupanagiota@yahoo.gr; 6 Division of Infectious Diseases, University General Hospital of Patras, 26504 Rio, Greece

**Keywords:** antimicrobial resistance, ICU, COVID-19, multidrug-resistant pathogens, bloodstream infections

## Abstract

The COVID-19 pandemic redirected infection control and antimicrobial stewardship towards SARS-CoV-2, potentially increasing multidrug-resistant (MDR) bloodstream infections (BSIs). We compared MDR BSI epidemiology and outcomes in a non-COVID intensive care unit (ICU) before and after the pandemic. The MATURATE ICU study retrospectively included adults with MDR BSIs admitted to a non-COVID ICU during the pre-COVID-19 (1 January 2017–1 January 2020) and post-COVID-19 (1 January 2022–1 January 2025) eras. Clinical, microbiological and outcome data were compared. Survival analyses and multivariable logistic regression identified predictors of ICU mortality. Overall, 146 patients were included (74 pre-COVID-19 and 72 post-COVID-19). Pre-COVID-19 patients were older and had higher comorbidity burden and severity indices (all *p* ≤ 0.023). ICU stay after the first positive blood culture was longer in the post-COVID-19 period (*p* = 0.003). New organ dysfunction, acute kidney injury, and septic shock were more frequent pre-COVID-19, whereas cardiovascular events were more frequent post-COVID-19. ICU mortality was higher pre-COVID-19 (*p* < 0.001). Competing-risk analysis, treating ICU discharge alive as a competing event, demonstrated a significantly lower cumulative incidence of ICU death in the post-COVID-19 era (adjusted sHR 0.20, 95% CI 0.11–0.35; *p* < 0.001). In multivariable analysis, post-COVID-19 era independently predicted lower 28-day ICU mortality (adjusted OR = 0.11, 95% CI 0.04–0.28, *p* < 0.001), whereas higher CCI (adjusted OR = 1.31, 95% CI 1.04–1.69, *p* = 0.027) and SOFA (adjusted OR = 1.22, 95% CI 1.07–1.40, *p* = 0.003) independently predicted increased mortality. Despite the persistently high burden of antimicrobial resistance, improved post-COVID-19 survival suggests that, advances in ICU care and healthcare system recovery may have contributed to better outcomes.

## 1. Introduction

Antimicrobial resistance (AMR) is an escalating global public health threat, with an estimated 1.14 million deaths directly attributable to AMR in 2021 (4.71 million deaths associated with AMR overall), rising to 1.91 million and 8.22 million deaths, respectively, by 2050 [1]. The COVID-19 pandemic further exacerbated the global AMR burden, with accumulating evidence indicating an increased prevalence of antimicrobial resistance among bacterial pathogens [2,3]. The widespread use of antibiotics, prolonged hospitalizations, increased intensive care unit (ICU) admissions, and the high incidence of secondary bacterial infections during the pandemic may have contributed to these trends [4]. Bloodstream infections (BSIs) represent a major focus in assessing the impact of the COVID-19 era on AMR [4], with recent meta-analyses reporting a pooled BSI prevalence of 8.2% among hospitalized COVID-19 patients, increasing in ICU settings [4,5], and a high prevalence of methicillin-resistant *Staphylococcus aureus* (MRSA) and extended-spectrum β-lactamase (ESBL)-producing *Enterobacterales* [6,7]. In addition, the emergence and dissemination of carbapenem-resistant organisms (CROs) have accelerated, particularly in ICU settings [8], whereas other pathogens, including *Candida auris*, also showed a marked increase during the COVID-19 pandemic, with reported cases rising nearly fivefold [9].

Similarly, data from Greece demonstrated an increasing incidence of resistant Gram-negative bacteria during the COVID-19 pandemic, particularly in ICUs, accompanied by increased prior antimicrobial use and higher rates of hospital-acquired infections (HAIs) [10,11]. Moreover, the post-pandemic period has been characterized by an increasing prevalence of infections caused by difficult-to-treat pathogens, including carbapenemase-producing *Enterobacterales* (CPE) (particularly KPC- and NDM-producing isolates), carbapenemase-producing *Pseudomonas aeruginosa* and fluconazole-resistant *Candida parapsilosis* [12,13,14]. These trends were also reflected in BSIs, where carbapenem-resistant *Klebsiella pneumoniae* exhibited a dynamic epidemiological shift during and after the COVID-19 pandemic, characterized by a declining predominance of KPC-producing isolates and a steady increase in metallo-β-lactamase (particularly NDM) and dual-carbapenemase-producing strains [15].

Although COVID-19 has been consistently associated with an increased burden of multidrug-resistant (MDR) BSIs in ICU patients [16,17], most available evidence originates from patients with SARS-CoV-2 infection or COVID-designated ICUs. In contrast, the impact of the pandemic on antimicrobial resistance in non-COVID ICU settings is likely to be more complex, as it reflects the combined effects of several, often opposing, pandemic-related changes in healthcare systems. While factors including increased antibiotic consumption and prolonged ICU stays may have promoted the emergence of MDR pathogens [18,19], enhanced infection prevention and control measures implemented during the pandemic would be expected to limit their transmission [20]. In parallel with these opposing pandemic-related effects, important advances have also been made in the early diagnosis and management of sepsis and infections caused by MDR pathogens. The Surviving Sepsis Campaign has placed greater emphasis on the early recognition of sepsis by recommending more sensitive screening strategies for hospitalized patients with suspected infection [21]. Furthermore, recent molecular diagnostic tests have substantially improved the rapid microbiological diagnosis of BSIs by enabling earlier identification of causative pathogens and AMR determinants compared with conventional blood cultures [22,23]. From a therapeutic perspective, updated international guidance, including the 2024 and recently updated 2026 Infectious Diseases Society of America (IDSA) guidance and the European Society of Clinical Microbiology and Infectious Diseases (ESCMID) recommendations (available at the post-COVID-19 time), has expanded therapeutic options for MDR Gram-negative infections through the incorporation of novel β-lactam/β-lactamase inhibitor combinations, including ceftazidime-avibactam, meropenem-vaborbactam and imipenem-cilastatin-relebactam, for the treatment of carbapenem-producing organisms. In addition, these guidelines recommend cefiderocol and the combination of aztreonam-avibactam for infections caused by metallo-β-lactamase-producing pathogens, while also incorporating sulbactam-durlobactam as the preferred treatment for carbapenem-resistant *Acinetobacter baumannii* infections [24,25,26]. Overall, critical care medicine continued to evolve throughout this period, with increasing experience in advanced organ support techniques such as extracorporeal membrane oxygenation (ECMO) [27], updated evidence-based management of acute respiratory distress syndrome (ARDS) [28], and the integration of artificial intelligence and digital technologies into intensive care practice [29]. Consequently, data comparing non-COVID ICUs before and after the COVID-19 pandemic remain limited, while data specifically addressing BSI caused by multidrug-MDR pathogens are even more scarce. Therefore, the present study aimed to compare the epidemiology, microbiological characteristics, and clinical outcomes of MDR BSIs among critically ill patients admitted to a non-COVID ICU during the pre-COVID-19 and post-COVID-19 eras.

## 2. Materials and Methods

### 2.1. Study Design

The MATURATE ICU study is a single-center retrospective observational study including adult patients with MDR BSIs admitted to the non-COVID ICU of a tertiary hospital in Greece. Patients were classified according to the period of admission into two groups: the pre-COVID-19 era (1 January 2017–1 January 2020) and the post-COVID-19 era (1 January 2022–1 January 2025). Demographic, clinical, microbiological, antimicrobial resistance, and outcome data were retrospectively collected and compared between the two periods.

### 2.2. Study Sample

The study sample consisted of consecutive adult patients (≥18 years) admitted to the non-COVID ICU of a tertiary university hospital during two predefined study periods: the pre-COVID-19 era (January 2017–January 2020) and the post-COVID-19 era (January 2022–January 2025). Because this was an exploratory retrospective cohort including all consecutive eligible patients, no formal sample size calculation was performed. Patients with a first documented episode of BSI caused by a MDR bacterial or fungal pathogen were considered eligible for inclusion.

For the purposes of the present study, only the first episode of BSI occurring within the study period was included in the analysis. In patients with repeated isolation of the same microorganism, only the first isolate was considered.

Blood cultures yielding microorganisms commonly regarded as skin commensals (e.g., coagulase-negative staphylococci, *Corynebacterium* spp., and *Bacillus* spp.) were considered clinically significant only when at least two blood cultures yielded the same microorganism with identical antimicrobial susceptibility patterns or when clinical and microbiological findings supported a true BSI. Each case involving potential skin contaminants was independently reviewed by an infectious diseases specialist before inclusion. Patients with contaminants that did not meet the predefined inclusion criteria, duplicate isolates, or missing essential clinical or microbiological data (including dates of admission, BSI, discharge or death, pathogen identification, antimicrobial susceptibility results, or treatment details) were excluded from the analysis. MDR-BSI onset was defined as the date of the first blood culture yielding an MDR pathogen (first MDR-positive blood culture).

### 2.3. Definitions

Bacterial isolates were classified as MDR, extensively drug-resistant (XDR), or pandrug-resistant (PDR) according to the international consensus criteria proposed by Magiorakos et al. [30]. MDR isolates were resistant to at least one antimicrobial agent in three or more antimicrobial categories, whereas XDR isolates remained susceptible to no more than two antimicrobial categories. PDR isolates were resistant to all antimicrobial agents across all antimicrobial categories tested. Isolates that did not meet the internationally accepted criteria for MDR, XDR, or PDR were classified as susceptible.

As these bacterial MDR/XDR/PDR definitions cannot be directly applied to *Candida* spp. and no standard definition for MDR *Candida* has been established, we followed a previously proposed approach in the literature. Accordingly, MDR *Candida* was defined as nonsusceptibility to at least one agent in at least two antifungal drug classes, whereas XDR was defined as non-susceptibility to at least one agent in at least three antifungal drug classes [31].

Central line-associated BSI (CLABSI) was defined as a primary BSI occurring in a patient with a central venous catheter that had been in place for ≥48 h before the onset of the bloodstream infection, with no evidence of infection at another site that could account for the BSI [32].

### 2.4. Data Collection

Clinical, demographic, and microbiological data were retrospectively obtained from the hospital’s electronic medical records, microbiology laboratory information system, and archived patient charts. Recorded demographic and baseline clinical variables included age, sex, anthropometric measurements, medical history and comorbidities, reason for hospital and ICU admission, medications received before and during hospitalization, previous antimicrobial exposure, need for mechanical ventilation and tracheostomy, and the duration of hospital and ICU stay. Data on mortality and the timing of death were also collected. The outcome of interest was ICU mortality, defined as death occurring during the ICU stay. For the 28-day logistic regression analysis, 28-day ICU mortality was defined as death occurring in the ICU within 28 days of the first MDR-positive blood culture; patients discharged alive from the ICU before day 28 were considered not to have experienced the predefined outcome. Comorbidity burden was assessed using the Charlson Comorbidity Index (CCI) [33], whereas acute clinical severity was evaluated using the Sequential Organ Failure Assessment (SOFA) score [34], Acute Physiology and Chronic Health Evaluation II (APACHE II) score [35], Pitt bacteremia score [36], and Systemic Inflammatory Response Syndrome (SIRS) criteria [37]. Laboratory findings obtained on the date of the first MDR positive blood culture were also recorded. When the same microorganism was isolated repeatedly from the same patient, only the first isolate associated with the first BSI episode was included in the primary analysis.

Microbiological variables included the organism identified in the first MDR-positive blood culture, its antimicrobial susceptibility profile and resistance phenotype, the presumed source of BSI, the presence of polymicrobial BSI, and any subsequent MDR isolates recovered during hospitalization. The intervals from hospital and ICU admission to the first MDR-positive blood culture, from blood culture collection to the initiation of appropriate antimicrobial therapy, and from the first positive culture to microbiological clearance or death were documented.

### 2.5. Pathogen Identification

Blood cultures were processed according to the routine workflow of the hospital’s Clinical Microbiology Laboratory. Pathogen identification and antimicrobial susceptibility testing were performed using the VITEK^®^ 2 automated system (bioMérieux, Marcy-l’Étoile, France) with the appropriate identification and susceptibility cards for Gram-positive bacteria, Gram-negative bacteria, and yeasts. Antimicrobial susceptibility results were interpreted according to the European Committee on Antimicrobial Susceptibility Testing (EUCAST) recommendations using the Clinical Breakpoint Tables version 16.0 (2026) [38]. Isolates were categorized as susceptible (including the susceptible, increased exposure category) or resistant according to EUCAST criteria. Minimum inhibitory concentrations (MICs) for selected antimicrobial agents were also determined. Colistin susceptibility was confirmed using the broth microdilution method (SensiTest™ Colistin, Liofilchem, Roseto degli Abruzzi, Italy), in accordance with current international recommendations. ESBL production was recorded according to the microbiology laboratory’s classification, with isolates reported as ESBL-positive classified accordingly.

### 2.6. Statistical Analysis

Categorical variables were summarized as frequencies and percentages, whereas continuous variables were expressed as medians and interquartile ranges (IQRs). Associations between categorical variables were assessed using Pearson’s chi-square test or Fisher’s exact test, as appropriate. Normality was evaluated using the Shapiro–Wilk and Kolmogorov–Smirnov tests. Continuous variables were compared using Student’s *t*-test or the Mann–Whitney U test according to their distribution.

Because ICU discharge alive precludes subsequent ICU death, it was considered a competing event. Cumulative incidence functions (CIFs) were estimated and compared using Gray’s test. Fine–Gray competing-risk regression was subsequently applied to evaluate independent predictors of ICU mortality after adjustment for clinically relevant covariates. Additional sensitivity analyses further adjusted for hospital-acquired infection (HAI) status, timing of BSI relative to ICU admission, and polymicrobial infection to assess the robustness of the primary competing-risk findings to additional clinically relevant covariates. Cause-specific Cox proportional hazards models were additionally performed. Kaplan–Meier survival curves, compared using the log-rank test, and univariable Cox proportional hazards regression were retained as complementary conventional time-to-event analyses. The proportional hazards assumption was assessed using Schoenfeld residuals.

As a secondary analysis, 28-day ICU mortality was evaluated using univariable and multivariable logistic regression models, with results reported as odds ratios (ORs) and 95% confidence intervals (CIs).

A sensitivity analysis was performed by excluding patients with an MDR-positive blood culture at ICU admission and repeating the primary analyses. All tests were two-tailed, and statistical significance was set at *p* = 0.05. All effect estimates are presented with 95% CI. All analyses and figure generation were performed in R (version 4.5.2) using RStudio 2026.01.1—Build 403 (PBC, Boston, MA, USA). The following R packages were used: broom v1.0.12, car v3.1-5, cmprsk v2.2-12, dplyr v1.2.0, epiDisplay v3.7.0.0, flextable v0.9.11, ggplot2 v4.0.2, Greg v2.0.3, gtsummary v2.5.0, janitor v2.2.1, nortest v1.0-4, pROC v1.19.0.1, rcompanion v2.5.2, ResourceSelection v0.3-6, survival v3.8-6, and survminer v0.5.2.

### 2.7. Ethics

The study protocol was reviewed and approved by the Research Ethics Committee and the Institutional Review Board of the University General Hospital of Patras (Approval No. 533/03.10.2023). Given the retrospective observational design of the study and the use of anonymized routinely collected clinical data, the requirement for informed consent was waived by the Ethics Committee. The study was conducted in accordance with Good Clinical Research Practice principles and the Declaration of Helsinki.

## 3. Results

### 3.1. Sample Characteristics

In total, 146 patients with MDR BSIs admitted to the non-COVID ICU were included in the study, of whom 74 (50.7%) were admitted during the pre-COVID-19 era and 72 (49.3%) during the post-COVID-19 era (Table 1). Regarding vaccination history, vaccination status was more frequently undocumented in the pre-COVID-19 era than in the post-COVID-19 era, for both influenza (52.7% vs. 22.2%, *p* < 0.001) and pneumococcal (54.1% vs. 27.8%, *p* < 0.001) vaccination. Patients in the pre-COVID-19 era were significantly older than those in the post-COVID-19 era (median age, 62.5 vs. 50 years, *p* < 0.001) and had a higher comorbidity burden, as reflected by higher CCI scores (4 vs. 1, *p* < 0.001) The proportion of patients who developed a hospital-acquired infection during their hospitalization was significantly higher in the pre-COVID-19 era than in the post-COVID-19 era (41.9% vs. 20.8%, *p* = 0.010). No significant differences were observed between the two groups regarding body mass index, sex, history of smoking, surgery, or the need for invasive airway support. Similarly, the distribution of the primary source of infection, including urinary tract, respiratory tract, central venous catheter (CVC)-related, hepatobiliary, central nervous system, gastrointestinal, soft tissue, trauma-related infections, osteomyelitis, and endocarditis, did not differ significantly between the two eras.

Patients in the pre-COVID-19 era presented with significantly greater illness severity at the onset of MDR BSI. Median SOFA scores were significantly higher than those observed in the post-COVID-19 era (10 vs. 7, *p* < 0.001), accompanied by higher APACHE II scores (25 vs. 18, *p* < 0.001) and a greater number of SIRS criteria fulfilled (3 vs. 2, *p* = 0.023).

Several laboratory parameters differed significantly between the two study periods. Compared with patients admitted during the post-COVID-19 era, those in the pre-COVID-19 period had significantly lower platelet counts (208,006 vs. 275,250/mm^3^, *p* < 0.001), higher serum creatinine (1.05 vs. 0.65 mg/dL, *p* < 0.001) and urea concentrations (86.5 vs. 41 mg/dL, *p* < 0.001), as well as, lower serum albumin (2.6 vs. 3.2 g/dL, *p* < 0.001) and total protein levels (5.2 vs. 5.7 g/dL, *p* = 0.003). Conversely, alanine aminotransferase (ALT) levels were significantly higher in the post-COVID-19 era (48 vs. 30 IU/L, *p* = 0.019). White blood cell count, neutrophil and lymphocyte percentages, hemoglobin concentration, international normalized ratio (INR), total bilirubin, aspartate aminotransferase (AST), and C-reactive protein (CRP) levels were comparable between the two groups.

**Table 1:** Sample characteristics.

	Era		
	Pre COVID-19 (2017–2020), *n* = 74	Post COVID-19 (2022–2025), *n* = 72	*p*
*Epidemiology*			
Age, years	62.5 (53.2–74.8)	50 (38.5–64.5)	<0.001
BMI, kg/m^2^	26.8 (24.6–29.4)	26.9 (24.2–31)	0.875
Male sex, *n* (%)	45 (60.81%)	43 (59.72%)	1
Smoking exposure, pack-years	0 (0–40)	0 (0–45)	0.942
Surgery (any time), *n* (%)	25 (33.78%)	32 (44.44%)	0.250
CCI	4 (2–5.75)	1 (0–4)	<0.001
Hospital-acquired infection, *n* (%)	31 (41.89%)	15 (20.83%)	0.010
Invasive airway support (intubation and/or tracheostomy), *n* (%)	55 (74.32%)	56 (77.78%)	0.768
*Diagnosis (source of infection)*			
UTI, *n* (%)	4 (5.41%)	2 (2.78%)	0.681
RTI, *n* (%)	25 (33.78%)	17 (23.61%)	0.240
CVC-related, *n* (%)	5 (6.76%)	0	0.058
Hepatobiliary, *n* (%)	3 (4.05%)	2 (2.78%)	1
CNS, *n* (%)	6 (8.11%)	6 (8.33%)	1
GI, *n* (%)	9 (12.16%)	9 (12.50%)	1
Endocarditis, *n* (%)	1 (1.35%)	0	1
Soft tissue, *n* (%)	5 (6.76%)	4 (5.56%)	1
Trauma, *n* (%)	13 (17.57%)	19 (26.39%)	0.276
Osteomyelitis, *n* (%)	1 (1.35%)	0	1
*Severity indices*			
SOFA score	10 (8–13)	7 (5–11)	<0.001
SIRS criteria	3 (2–3)	2 (1–3)	0.023
APACHE II score	25 (20.2–30)	18 (14–21)	<0.001
*Laboratory values*			
WBC, /mm^3^	12,895 (8375–18,145)	12,305 (8255–17,192)	0.920
Neutrophils, %	84 (76.6–90.9)	81.2 (73.8–89.9)	0.210
Lymphocytes, %	7.55 (4.6–11.9)	9.3 (5.7–13.4)	0.195
Hemoglobin, g/dL	9.2 (8.33–10.1)	9.65 (8.7–10.9)	0.100
Platelets, /mm^3^	208,006 (106,750–255,750)	275,250 (188,250–356,000)	<0.001
INR	1.17 (1.09–1.31)	1.16 (1.07–1.28)	0.241
Creatinine, mg/dL	1.05 (0.7–2.18)	0.65 (0.575–0.9)	<0.001
Urea, mg/dL	86.5 (46.5–136)	41 (29.8–68.5)	<0.001
Total bilirubin, mg/dL	0.745 (0.51–1.55)	0.75 (0.41–1.11)	0.451
AST (SGOT), IU/L	43 (29–99.2)	39.5 (28–74.2)	0.512
ALT (SGPT), IU/L	30 (19–76.8)	48 (29.5–89)	0.019
Albumin, g/dL	2.6 (2.3–3)	3.2 (2.77–3.5)	<0.001
Total protein, g/dL	5.2 (4.6–5.8)	5.7 (5–6.2)	0.003
CRP, mg/dL	9.81 (4.49–16.2)	7.88 (4.43–16.3)	0.668

Abbreviations: BMI, body mass index; CCI, Charlson Comorbidity Index; UTI, urinary tract infection; RTI, respiratory tract infection; CVC, central venous catheter; CNS, central nervous system; GI, gastrointestinal; SOFA, Sequential Organ Failure Assessment; SIRS, Systemic Inflammatory Response Syndrome; APACHE II, Acute Physiology and Chronic Health Evaluation II; WBC, white blood cell; INR, international normalized ratio; AST, aspartate aminotransferase; ALT, alanine aminotransferase; SGOT, serum glutamic-oxaloacetic transaminase; SGPT, serum glutamic-pyruvic transaminase; CRP, C-reactive protein.

### 3.2. BSI Characteristics

The characteristics of BSI episodes are summarized in Table 2. Patients in the pre-COVID-19 era had significantly higher Pitt bacteremia scores than those in the post-COVID-19 era (median 8 vs. 6, *p* = 0.001), indicating greater disease severity at the onset of MDR BSI. In contrast, patients admitted during the post-COVID-19 era remained in the ICU for a significantly longer period following the first MDR-positive blood culture (median 29 vs. 18 days, *p* = 0.003). No significant differences were observed in the time from hospital admission to the first MDR-positive blood culture, the time from ICU admission to the first MDR-positive blood culture, or the interval between the first MDR-positive blood culture and initiation of appropriate antimicrobial therapy (all *p* > 0.05). The distribution of CVC insertion sites differed significantly between the two eras (*p* = 0.006), with the jugular vein representing the predominant insertion site in both groups, whereas femoral and peripherally inserted central catheter (PICC) placement were more common in the post-COVID-19 era and subclavian catheterization was more frequently observed in the pre-COVID-19 era.

### 3.3. Pathogen-Related Characteristics

Although not statistically significant, polymicrobial infections were more frequent in the post-COVID-19 era (Table 3). There were no statistically significant differences in pathogen category (Gram-positive, Gram-negative, or fungi) or primary pathogen distribution among the included MDR-BSI cases between eras (Table 3). The XDR classification remained stable between eras, and no isolate satisfied the criteria for PDR in either era (Table 3). The resistance mechanisms/phenotypes among the assessable isolates are shown in Table 3 and were comparable between eras. Second and third pathogens are summarized in Supplementary Table S1.

### 3.4. Complications and Outcomes

Clinical outcomes are summarized in Table 4. Patients in the pre-COVID-19 era experienced significantly higher rates of new organ dysfunction during ICU stay (89.19% vs. 54.17%, *p* < 0.001), acute kidney injury (AKI) (68.92% vs. 29.17%, *p* < 0.001), and septic shock (67.57% vs. 40.28%, *p* = 0.001) compared with those admitted during the post-COVID-19 era. In contrast, acute coronary syndrome/myocardial infarction occurred more frequently in the post-COVID-19 era (9.72% vs. 1.35%, *p* = 0.032). No significant differences were observed in the incidence of ARDS, disseminated intravascular coagulation (DIC), or the time from initiation of active antimicrobial therapy to the first negative blood culture. ICU mortality was markedly higher among patients admitted during the pre-COVID-19 era (83.8% vs. 25.0%, *p* < 0.001), whereas the overall ICU length of stay was significantly longer in the post-COVID-19 era (median 44 vs. 31 days, *p* = 0.012).

### 3.5. Competing-Risk and Survival Analyses

**Figure 1:**
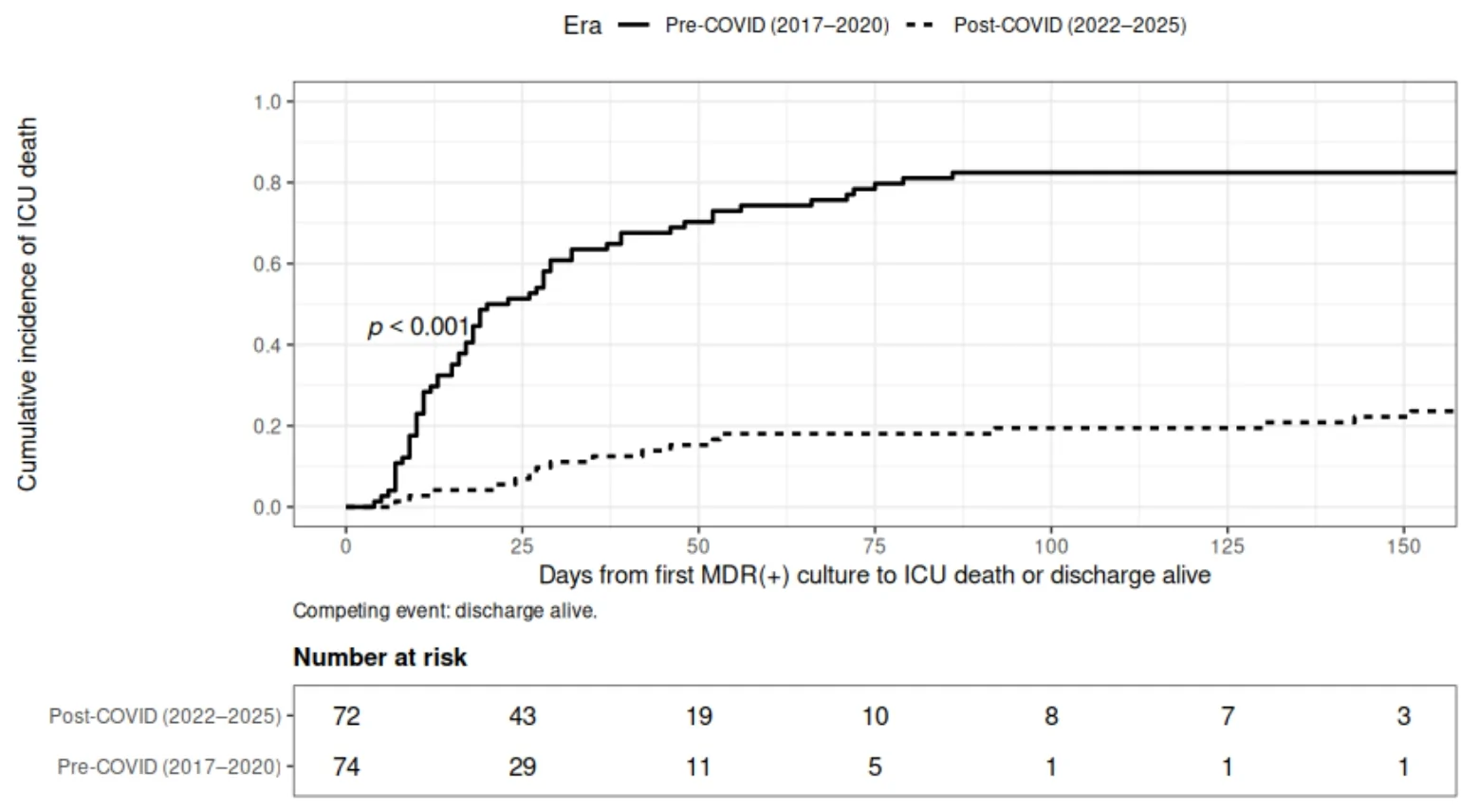
CIF of ICU death by era, with discharge alive treated as a competing event. Time zero was the first MDR-positive blood culture. Curves are shown up to 150 days for visual clarity, along with the number at risk at each time point. CIFs were compared between eras using Gray’s test.

Because ICU discharge alive represents a competing event for ICU death, cumulative incidence functions (CIFs) were used as the primary approach to estimate the cumulative incidence of ICU death. The cumulative incidence of ICU death differed significantly between the two study periods (Gray’s test, *p* < 0.001) (Figure 1). In multivariable Fine–Gray regression adjusted for age, CCI, and SOFA score, the post-COVID-19 era remained strongly associated with a lower subdistribution hazard of ICU death (sHR = 0.20, 95% CI 0.11–0.35; *p* < 0.001; Table 5). The association between the post-COVID-19 era and the subdistribution hazard of ICU death remained stable in sensitivity analyses additionally adjusting for HAI status, BSI timing, and polymicrobial infection (sHR range 0.19–0.20; all *p* < 0.001). The median time from the first MDR-positive blood culture to ICU discharge was 24 days (IQR, 12–43), with a maximum observed follow-up of 257 days.

**Figure 2:**
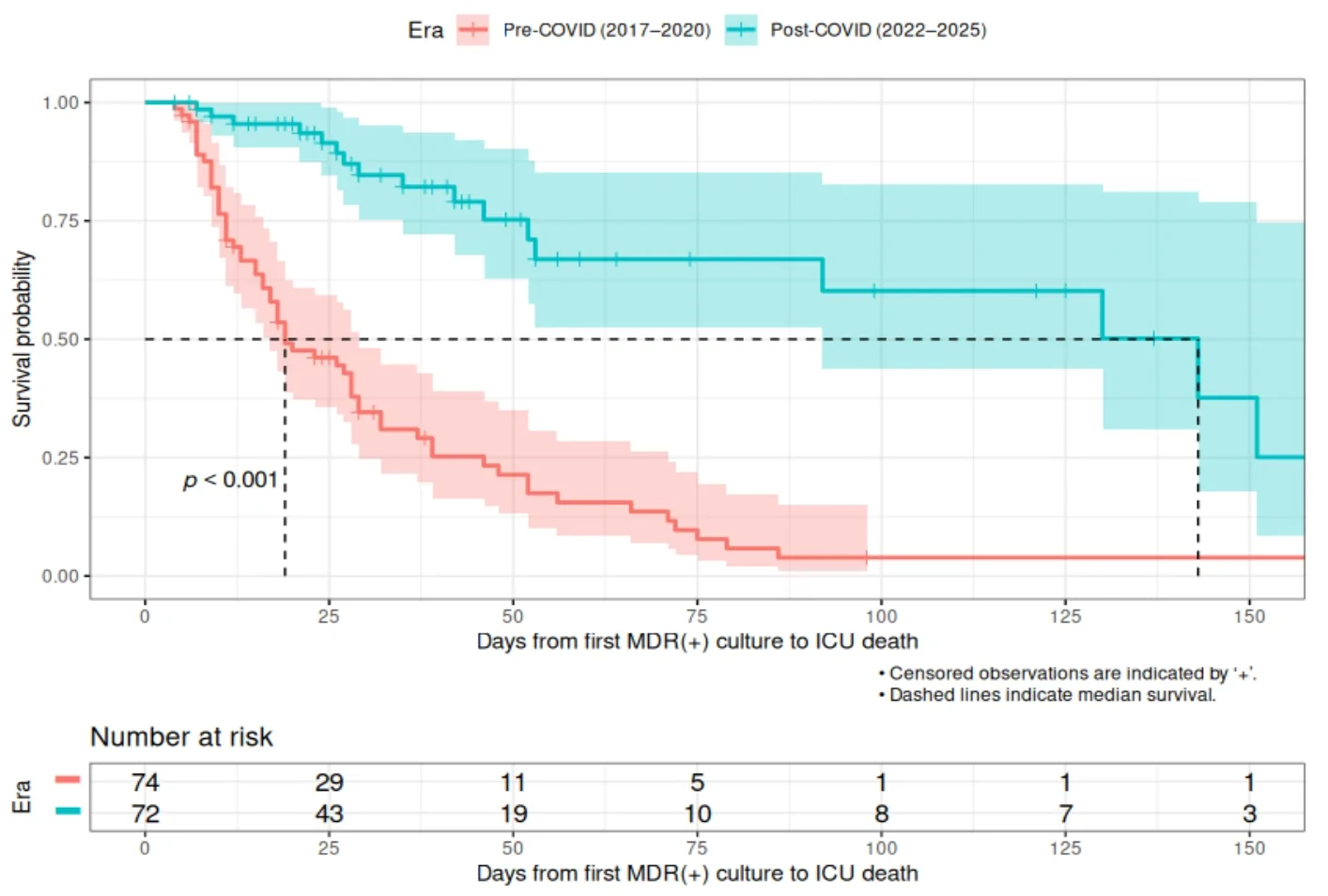
Kaplan–Meier survival curves for ICU mortality following MDR onset, stratified by era (pre-COVID-19 vs. post-COVID-19).

Complementary Kaplan–Meier analysis showed a significant difference in ICU survival after the first MDR-positive culture between the pre- and post-COVID-19 eras (log-rank χ^2^ = 44.6, *p* < 0.001) (Figure 2). Median ICU survival from MDR onset was 19 days (95% CI 17–29) in the pre-COVID-19 group and 143 days (95% CI 92–NE) in the post-COVID-19 group. Similar findings were observed when time was measured from ICU admission (log-rank χ^2^ = 40.5, *p* < 0.001), with median ICU survival of 34 days (95% CI 30–49) and 152 days (95% CI 92–NE), respectively.

Univariable Cox regression yielded concordant results, with lower hazards of ICU death in the post-COVID-19 era whether time was measured from ICU admission (HR 0.208, 95% CI 0.122–0.354; *p* < 0.001) or from MDR onset (HR 0.193, 95% CI 0.113–0.329; *p* < 0.001).

### 3.6. Multivariable Cause-Specific Cox

In multivariable cause-specific Cox regression for ICU death (time zero defined as the first MDR-positive culture; ICU discharge alive treated as censoring), the post-COVID-19 era was associated with a markedly lower hazard of ICU death compared with the pre-COVID-19 era (HR 0.23, 95% CI 0.13–0.40; *p* < 0.001), after adjustment for age, CCI, and SOFA score. A higher SOFA score (HR 1.12 per point, 95% CI 1.05–1.20; *p* = 0.001) and a higher CCI (HR 1.15 per point, 95% CI 1.02–1.29; *p* = 0.022) were independently associated with increased ICU mortality risk. However, age was not associated with this risk (*p* = 0.53). The proportional hazards assumptions were assessed using Schoenfeld residuals and were found to be satisfied (global test *p* = 0.41).

In a separate cause-specific Cox model for ICU discharge alive (ICU death treated as censoring), the post-COVID-19 period was associated with a higher rate of discharge compared with the pre-COVID-19 period (HR 2.35, 95% CI 1.21–4.55; *p* = 0.011). Overall, the proportional hazards diagnostics demonstrated acceptable validity (global test *p* = 0.20). A borderline time-varying effect was indicated for age (*p* = 0.046), while the era effect satisfied the proportional hazards assumption (*p* = 0.52). In practical terms, the post-COVID-19 period was characterized by a substantially lower mortality hazard and a higher discharge hazard following MDR onset in comparison with the pre-COVID-19 period.

### 3.7. 28-Day ICU Mortality—Logistic Regression Analysis

The post-/pre-COVID-19 era, age, CCI, and SOFA score at the time of the index MDR-positive blood culture were evaluated for their association with 28-day ICU mortality from MDR BSI onset (death within 28 days ICU stay from the first MDR-positive blood culture). In univariate analysis, the post-COVID-19 era was associated with markedly reduced odds of 28-day ICU death compared with the pre-COVID-19 era (OR = 0.08, 95% CI: 0.03–0.18, *p* < 0.001). Higher CCI (OR = 1.32, 95% CI: 1.15–1.54, *p* < 0.001) and higher SOFA score (OR = 1.27, 95% CI: 1.14–1.42, *p* < 0.001) were also significantly associated with increased odds of death. Age was associated with mortality in the univariate model (OR = 1.03 per year, 95% CI: 1.01–1.05, *p* = 0.009).

In the multivariable logistic regression model, which was adjusted for age, CCI, and SOFA score, the post-COVID-19 era remained independently associated with substantially lower odds of 28-day ICU mortality (adjusted OR = 0.11, 95% CI: 0.04–0.28, *p* < 0.001). The results of the analysis indicated that both CCI (adjusted OR = 1.31 per point, 95% CI: 1.04–1.69, *p* = 0.027) and SOFA score (adjusted OR = 1.22 per point, 95% CI: 1.07–1.40, *p* = 0.003) continued to be statistically significant predictors. However, the analysis revealed that age was no longer a significant factor after adjustment (adjusted OR = 0.98 per year, 95% CI: 0.95–1.02, *p* = 0.284). The unadjusted and adjusted results are summarized in Table 6.

To account for a small number of patients with an MDR-positive culture on the day of ICU admission (n = 19; 12 in the pre-COVID-19 era and 7 in the post-COVID-19 era), all key analyses were repeated after excluding these cases. The findings were consistent with the persistent improvement in post-COVID-19 outcomes observed across Kaplan–Meier/log-rank and competing-risks analyses. Additionally, similar adjusted effect estimates were identified in the Fine–Gray, cause-specific Cox (death and discharge), and the 28-day logistic model.

## 4. Discussion

The present study aimed to compare the epidemiology, clinical characteristics, and outcomes of MDR BSIs among critically ill patients admitted to a non-COVID ICU before and after the COVID-19 pandemic. We found that, despite comparable pathogen distribution and AMR profiles between the two study periods, patients admitted during the post-COVID-19 era experienced substantially lower ICU mortality following MDR-BSI onset. Although patients in the post-pandemic cohort remained hospitalized for longer after MDR BSI onset, they developed fewer sepsis-related complications, including AKI, septic shock, and new organ dysfunction. These findings remained robust across multiple complementary analytical approaches, including competing-risk regression, cause-specific Cox proportional hazards models, Kaplan–Meier survival analysis, multivariable logistic regression, and sensitivity analyses, suggesting that the observed survival benefit was unlikely to be explained solely by differences in patient characteristics or analytical methodology. Collectively, these findings suggest that, in endemic MDR settings, determinants beyond microbiological resistance—including healthcare-system recovery, improvements in ICU organization, advances in supportive care, and optimization of antimicrobial management—may exert a greater influence on patient outcomes than pathogen resistance alone.

We found that, patients admitted to the ICU during the pre-COVID-19 era were older, had a significantly higher burden of comorbidities, and presented with greater severity indices such as SOFA, Pitt bacteremia and APACHE II score than those admitted after the pandemic. These differences were further supported by laboratory parameters indicative of advanced systemic illness, including thrombocytopenia, hypoalbuminemia, hypoproteinemia, and impaired renal function. These findings describe a population with significantly reduced physiological reserve and greater susceptibility to sepsis-related organ dysfunction. Previous investigations have consistently identified both chronic comorbidity burden and acute organ dysfunction as the strongest determinants of mortality among critically ill patients with BSI [39], frequently outweighing microbiological characteristics themselves. Likewise, hypoalbuminemia, thrombocytopenia and AKI have repeatedly been associated with impaired host immune response, endothelial dysfunction, excessive inflammatory activation and poor survival in septic patients [40,41,42]. Our findings therefore suggest that the two study groups differed not only chronologically but also biologically, reflecting distinct baseline risks at the time bloodstream infection developed.

The reasons underlying these differences are probably multifactorial. During the COVID-19 pandemic, healthcare systems underwent profound organizational changes, including ICU restructuring, redistribution of personnel, modification of referral pathways, postponement of elective procedures and alterations in admission criteria [43]. Although these measures were initially implemented to cope with the unprecedented burden of SARS-CoV-2, their effects extended well beyond the acute pandemic phase. Several studies have demonstrated persistent changes in ICU admission patterns following the pandemic, although the direction of these changes has varied considerably across healthcare systems [44,45]. While some cohorts reported progressively older and more severely ill ICU populations after COVID-19, others demonstrated recovery of routine critical care services with earlier referral and improved access to intensive care [46,47,48]. These discrepancies probably reflect differences in national healthcare organization, ICU capacity, referral networks and local epidemiology. Consequently, the younger age and lower illness severity observed in our post-pandemic cohort likely represent changes in healthcare delivery, rather than biological differences in MDR pathogens themselves. Importantly, however, baseline severity alone does not appear sufficient to explain the remarkable reduction in mortality observed after the pandemic. After adjustment for age, comorbidity burden and organ dysfunction, admission during the post-COVID era remained independently associated with substantially lower mortality across all analytical approaches, including multivariable logistic regression, cause-specific Cox regression and Fine-Gray competing-risk analysis. The remarkable consistency of these findings considerably strengthens the robustness of the observed association and argues against differences in baseline characteristics as the sole explanation. Although residual confounding cannot be completely excluded in any retrospective study, these findings suggest that additional healthcare-related factors likely contributed to the improved prognosis.

Additionally, no significant differences were observed in the microbiological epidemiology among the included MDR-BSI cases despite the major temporal changes in patient outcomes. The relative distribution of Gram-positive organisms, Gram-negative pathogens and fungi remained largely unchanged, as did the proportion of extensively drug-resistant isolates and the major resistance mechanisms, including carbapenem-resistant *A. baumannii*, carbapenem-resistant *Enterobacterales*, methicillin-resistant coagulase-negative staphylococci and vancomycin-resistant enterococci. This finding contrasts with numerous reports describing substantial microbiological shifts during the COVID-19 pandemic, particularly increasing prevalence of carbapenem-resistant Gram-negative organisms, multidrug-resistant *A. baumannii* and difficult-to-treat *K. pneumoniae* [49]. These changes have been largely attributed to the widespread empirical use of antibiotics during the COVID-19 pandemic, prolonged ICU hospitalization, increased use of invasive devices, and disruptions in infection prevention and antimicrobial stewardship programs [3,50]. However, most published studies compared pre-pandemic with pandemic periods or evaluated COVID-designated ICUs, whereas considerably fewer data are available regarding non-COVID ICUs following restoration of routine healthcare services. Our findings therefore suggest that, in endemic MDR settings such as Greece, the pandemic may have exerted a smaller long-term influence on pathogen ecology than previously anticipated [51,52,53].

The apparent microbiological stability observed in our cohort should also be interpreted within the broader epidemiological context of antimicrobial resistance in Greece. Unlike many European countries where resistance increased primarily during the pandemic, Greece has remained one of the countries with the highest AMR-burden in Europe for more than a decade. National surveillance has consistently demonstrated persistently high rates of carbapenem-resistant *K. pneumoniae*, multidrug-resistant *A. baumannii* and resistant non-fermenting Gram-negative bacilli, reflecting long-standing endemic circulation rather than transient pandemic-related outbreaks [51,52,53]. Consequently, the absence of major microbiological differences between the two study periods is biologically plausible and suggests that endemic resistance patterns may be considerably more stable than those observed in healthcare systems with lower baseline AMR prevalence. This observation further emphasizes that improvements in clinical outcomes may occur even in environments where pathogen resistance remains largely unchanged.

Interestingly, although the principal pathogen distribution remained unchanged, several epidemiological trends among the included MDR-BSI cases deserve consideration. Polymicrobial BSIs were numerically more frequent during the post-pandemic period, while secondary and tertiary bloodstream isolates demonstrated greater microbiological diversity, including the emergence of *C. auris*. Although these observations did not achieve statistical significance and therefore should be interpreted cautiously, they are consistent with contemporary reports describing increasingly complex polymicrobial infections among critically ill patients receiving prolonged invasive support, multiple courses of broad-spectrum antimicrobial therapy and repeated healthcare exposure [54]. Rather than simply reflecting greater microbiological diversity, polymicrobial BSIs have been proposed as surrogate markers of healthcare complexity and cumulative invasive interventions. Their increasing frequency despite improved survival further suggests that, modern ICU outcomes may increasingly depend on successful management of complex host–pathogen interactions rather than pathogen characteristics alone.

In this context, one noteworthy finding was the persistent predominance of *Staphylococcus epidermidis* throughout both study periods. Although coagulase-negative staphylococci have historically been regarded primarily as blood culture contaminants, they are now recognized as important opportunistic pathogens among critically ill patients with intravascular devices, prosthetic material and prolonged ICU exposure [49,55]. Biofilm formation, immune evasion and extensive antimicrobial resistance have transformed these organisms into major causes of device-associated BSI worldwide [56]. The relatively short interval between ICU admission and the first MDR-positive blood culture observed in our study, together with the widespread use of central venous catheters, likely contributed to this predominance. Importantly, rigorous microbiological inclusion criteria were applied in the present study, including exclusion of probable contaminants and independent review of potentially contaminated cultures by infectious diseases specialists, supporting the clinical relevance of these isolates.

Changes in catheter insertion practices between study periods also warrant consideration. Subclavian catheterization was more common before the pandemic, whereas femoral and peripherally inserted central catheters were used more frequently afterward. Historically, subclavian access has been considered the preferred insertion site because of lower catheter colonization rates, whereas femoral catheterization has generally been associated with increased infectious complications [57]. However, more recent randomized trials and large multicentre observational studies have questioned the independent contribution of insertion site after adjustment for catheter maintenance practices, insertion bundles and patient-related factors [58,59]. Consequently, although catheter insertion practices evolved during the study period, these differences should not be interpreted as causal determinants of BSI [60,61,62,63]. Instead, they probably reflect changing ICU practices, vascular access preferences and increasing use of ultrasound-guided catheterization following the pandemic [60,61,62,63].

Mortality improved despite stable pathogen distribution and unchanged resistance phenotypes (25.0% vs. 83.8%). This difference remained consistent across all survival analyses and persisted after adjustment for age, comorbidity burden, and disease severity. This suggests that determinants other than microbiology, including changes in patient selection, ICU organization, supportive care, antimicrobial stewardship, and advances in critical care management, may have contributed more substantially to outcome than pathogen resistance itself. Although MDR pathogens undoubtedly complicate empirical antimicrobial selection, delay administration of appropriate therapy and increase healthcare costs [64], the relationship between antimicrobial resistance and mortality is considerably more complex than often appreciated. Recent studies increasingly indicate that, mortality associated with MDR BSIs reflects the interaction between pathogen virulence, host susceptibility, illness severity and healthcare delivery rather than resistance mechanisms alone [65,66]. In critically ill patients, progression to multiple organ dysfunction syndrome, rather than microbiological persistence antimicrobial therapy has been initiated, ultimately determines prognosis. This concept is strongly supported by our findings, where mortality improved dramatically despite stable resistance profiles, suggesting that optimization of critical care may partially offset the adverse prognostic impact of AMR.

Several observations from the present study support this interpretation. First, patients admitted during the post-pandemic period experienced significantly lower rates of septic shock, AKI and new organ dysfunction, despite comparable microbiological characteristics. Organ dysfunction represents the final common pathway through which BSIs lead to death, and prevention or early reversal of organ failure remains the principal therapeutic objective in modern sepsis management [67,68,69]. Accordingly, the lower incidence of sepsis-related complications observed after the pandemic probably contributed substantially to the improved survival demonstrated in all adjusted analyses. Similar observations have been reported in recent multicentre sepsis cohorts, where mortality has progressively declined despite relatively stable pathogen distribution, largely reflecting advances in organ support, implementation of evidence-based sepsis bundles and improvements in critical care delivery rather than changes in antimicrobial susceptibility patterns [70].

No significant differences were observed in the interval from the first positive blood culture to administration of active antimicrobial therapy. This finding deserves particular emphasis because delayed initiation of effective antimicrobial treatment has consistently been identified as one of the strongest modifiable predictors of mortality in bloodstream infections and septic shock [71]. The comparable treatment intervals observed in both cohorts argue against earlier antimicrobial administration as the principal explanation for the observed survival benefit. Likewise, microbiological clearance occurred at similar rates in both study periods, further supporting the hypothesis that differences in antimicrobial activity were unlikely to account for the substantial reduction in mortality. Instead, improvements in supportive management following infection—including hemodynamic optimization, renal replacement therapy, lung-protective ventilation, nutritional support, and multidisciplinary critical care—may have contributed more substantially to patient survival than antimicrobial factors alone.

The persistence of the association between study period and mortality after adjustment for age, CCI and SOFA score further strengthens this hypothesis. Although patients admitted before the pandemic undoubtedly presented with substantially greater illness severity, admission during the post-COVID era remained independently associated with lower mortality across all multivariable models. Importantly, illness severity, quantified by the SOFA score, and chronic comorbidity burden remained independent predictors of mortality in both the cause-specific Cox and logistic regression models, confirming previous evidence identifying these variables as major prognostic determinants among critically ill patients with BSIs [72,73]. Nevertheless, adjustment for these established risk factors did not eliminate the protective association of the post-pandemic period, indicating that additional, unmeasured factors probably contributed to the improved outcomes.

These findings challenge the traditional assumption that antimicrobial resistance represents the dominant determinant of outcome among critically ill patients with BSIs. Although AMR undoubtedly complicates therapeutic decision-making and increases healthcare costs, accumulating evidence indicates that host factors—including physiological reserve, organ dysfunction, immune dysregulation, timely source control and quality of supportive critical care—often exert a greater influence on prognosis once appropriate antimicrobial therapy has been initiated. Our observations strongly support this concept. Despite persistent circulation of highly resistant pathogens, patients admitted after the pandemic experienced substantially fewer episodes of septic shock, AKI and new organ dysfunction, suggesting that improvements in host management rather than changes in pathogen ecology largely accounted for the observed survival benefit.

This interpretation is further supported by the increasing recognition of healthcare-system resilience as a determinant of patient outcomes following the COVID-19 pandemic [74,75,76]. During the acute pandemic, unprecedented pressure on healthcare systems resulted in staff shortages, redeployment of inexperienced personnel, disruption of antimicrobial stewardship programmes, reduced adherence to infection prevention measures and delayed implementation of evidence-based sepsis pathways [74,75,76]. As healthcare systems progressively recovered, multidisciplinary ICU teams were re-established, antimicrobial stewardship programmes resumed, infection prevention practices strengthened and standardized critical care protocols became fully operational again [77]. Although these variables were not directly measured in the present study, they provide biologically plausible explanations for why mortality improved dramatically despite unchanged microbiological characteristics. Collectively, our findings therefore suggest that recovery of healthcare delivery may represent an underappreciated determinant of survival among patients with MDR BSIs.

The present study possesses several important strengths. To our knowledge, it represents one of the few investigations directly comparing MDR BSIs in a dedicated non-COVID ICU before and after the pandemic, while combining detailed microbiological characterization with multiple complementary survival methodologies. Inclusion of consecutive patients, standardized microbiological assessment according to contemporary EUCAST recommendations, comprehensive clinical characterization, adjustment for major confounders, competing-risk analyses, and confirmation of findings through sensitivity analyses collectively strengthen the internal validity of the study. Furthermore, evaluation of both microbiological and healthcare-related determinants provides a broader perspective on the complex factors influencing outcomes among critically ill patients with MDR BSIs.

Nevertheless, several limitations merit consideration. First, the retrospective, single-centre design inevitably introduces the possibility of selection bias, information bias and residual confounding despite comprehensive multivariable adjustment. Second, although all consecutive eligible patients were included, the relatively modest sample size may have limited statistical power for subgroup analyses, particularly regarding individual pathogens, resistance mechanisms and fungal BSIs. Third, several potentially important determinants of prognosis—including source-control procedures, appropriateness and optimization of antimicrobial therapy, therapeutic drug monitoring, timing of catheter removal, corticosteroid exposure, nutritional status, frailty and functional status before ICU admission—were not consistently available and therefore could not be incorporated into the adjusted models. Consequently, substantial residual confounding from these unmeasured factors cannot be excluded and may partly account for the observed association between the post-COVID-19 era and lower mortality. The interpretation of mortality outcomes also warrants caution, as mortality was assessed following MDR-BSI onset and should not be considered directly attributable to the MDR BSI itself. Fourth, the comparison between the pre- and post-COVID-19 eras represents a comparison of two distinct calendar periods and therefore cannot isolate the effect of the COVID-19 pandemic per se. The observed differences may also reflect changes occurring over time in ICU organization and staffing, patient referral and admission patterns, case mix, clinical practice, antimicrobial stewardship, diagnostic approaches, infection prevention measures, and treatment protocols. As these factors were not prospectively measured, their individual contribution to the observed differences could not be determined, precluding causal attribution to the pandemic itself. Furthermore, the study reflects the experience of a single tertiary referral centre located in a country with endemic AMR, and therefore extrapolation of these findings to regions with substantially different resistance epidemiology should be undertaken with caution. Moreover, because the study included only patients with documented MDR BSI, the findings describe the clinical and microbiological characteristics and pathogen distribution among MDR-BSI cases rather than the incidence, prevalence, or overall burden of MDR BSI in the ICU. Finally, due to the retrospective design of the study, robust epidemiological estimates, such as the incidence of overall and ICU-acquired MDR BSIs per 1000 patient-days, could not be calculated because the required denominator data were not available. Therefore, conclusions regarding the epidemiology of MDR BSIs should be interpreted with this limitation in mind.

## 5. Conclusions

In conclusion, critically ill patients with MDR BSIs admitted during the post-COVID-19 era experienced better survival than those admitted before the pandemic, despite the persistently high burden of AMR. The comparable distribution of MDR pathogens and resistance phenotypes across the two study periods suggests that improvements in clinical outcomes were not accompanied by a reduction in antimicrobial resistance. Our findings suggest that improving ICU outcomes in patients with MDR BSIs may depend as much on healthcare-system resilience, optimized supportive care, and stewardship as on controlling antimicrobial resistance itself.

## Figures and Tables

**Table 2:** Bacteremia-related characteristics.

	Era		
	Pre COVID-19 (2017–2020), *n* = 74	Post COVID-19 (2022–2025), *n* = 72	*p*
Pitt bacteremia score	8 (6–8.75)	6 (6–8)	0.001
Days from hospital admission to first positive culture	14 (6–27)	14 (8–19)	0.669
Days from ICU admission to first positive culture	6 (1–15)	10 (3.75–14.2)	0.185
First positive blood culture ≥ 2 days after ICU admission, *n* (%)	50 (67.6%)	59 (81.9%)	0.071
Days in ICU from first positive culture	18 (10.2–31.8)	29 (17.2–51.2)	0.003
Days from positive culture to start of active antibiotic	2 (0–3.5)	1 (0–3)	0.300
CVC site			0.006
Jugular, *n* (%)	56 (75.68%)	52 (72.22%)	
Femoral, *n* (%)	1 (1.35%)	7 (9.72%)	
Subclavian, *n* (%)	15 (20.27%)	5 (6.94%)	
PICC, *n* (%)	1 (1.35%)	5 (6.94%)	
No CVC, *n* (%)	1 (1.35%)	3 (4.17%)	

Abbreviations: ICU, intensive care unit; CVC, central venous catheter; PICC, peripherally inserted central catheter.

**Table 3:** Pathogen-related characteristics.

	Era		
	Pre COVID-19 (2017–2020), *n* = 74	Post COVID-19 (2022–2025), *n* = 72	*p*
Polymicrobial infection, *n* (%)	40 (54.05%)	49 (68.06%)	0.117
Pathogen category			0.867
Gram (+), *n* (%)	43 (58.11%)	44 (61.11%)	
Gram (−), *n* (%)	30 (40.54%)	27 (37.50%)	
Fungi, *n* (%)	1 (1.35%)	1 (1.39%)	
Primary pathogen identified			0.198
*S. epidermidis*, *n* (%)	31 (41.9%)	25 (34.7%)	
*K. pneumoniae*, *n* (%)	19 (25.7%)	11 (15.3%)	
*A. baumannii*, *n* (%)	10 (13.5%)	14 (19.4%)	
*S. hominis*, *n* (%)	5 (6.8%)	10 (13.9%)	
*E. faecium*, *n* (%)	1 (1.4%)	3 (4.2%)	
*S. haemolyticus*, *n* (%)	3 (4.1%)	1 (1.4%)	
*S. capitis*, *n* (%)	0 (0.0%)	3 (4.2%)	
Others, *n* (%)	5 (6.8%)	5 (6.9%)	
Extensively Drug-Resistant (XDR)			0.876
Yes, *n* (%)	17 (22.97%)	15 (20.83%)	
No, *n* (%)	55 (74.32%)	56 (77.78%)	
Not applicable, *n* (%)	2 (2.70%)	1 (1.39%)	
Bacterial resistance mechanisms			
CRAB, *n*/*N* (%)	8/10 (80.0%)	11/14 (78.6%)	1.000
ESBL, *n*/*N* (%)	18/18 (100%)	12/13 (92.3%)	0.655
CRE, *n*/*N* (%)	14/15 (93.3%)	9/11 (81.8%)	0.711
MR-CNS, *n*/*N* (%)	38/39 (97.4%)	37/37 (100%)	0.615
VRE, *n*/*N* (%)	1/1 (100%)	3/3 (100%)	1.000
Fungal resistant phenotype			
Azole resistance, *n*/*N* (%)	1/1 (100%)	0/1 (0%)	1.000
Echinocandin resistance, *n*/*N* (%)	1/1 (100%)	1/1 (100%)	1.000
Amphotericin B resistance, *n*/*N* (%)	0/1 (0%)	0/1 (0%)	1.000

1. XDR: *p*-values were calculated among isolates with available XDR classification; observations coded as “not applicable” were excluded. Resistance mechanisms/phenotypes: data are presented as n/N (%), where N includes only assessable isolates; observations coded as “not applicable” or “not accessed” were excluded from the denominator. 2. Comparisons involving resistance phenotypes with very small denominators are descriptive and should be interpreted with caution. 3. Abbreviations: XDR, extensively drug-resistant; *S. epidermidis*, *Staphylococcus epidermidis*; *K. pneumoniae*, *Klebsiella pneumoniae*; *A. baumannii*, *Acinetobacter baumannii*; *S. hominis*, *Staphylococcus hominis*; *E. faecium*, *Enterococcus faecium*; *S. haemolyticus*, *Staphylococcus haemolyticus*; *S. capitis*, *Staphylococcus capitis*; CRAB, carbapenem-resistant *Acinetobacter baumannii*; ESBL, extended-spectrum β-lactamase; CRE, carbapenem-resistant *Enterobacterales*; MR-CNS, methicillin-resistant coagulase-negative staphylococci; VRE, vancomycin-resistant enterococci.

**Table 4:** Complications and outcomes.

	Era		
	Pre COVID-19 (2017–2020), *n* = 74	Post COVID-19 (2022–2025), *n* = 72	*p*
New organ dysfunction during ICU stay, *n* (%)	66 (89.19%)	39 (54.17%)	<0.001
ARDS, *n* (%)	15 (20.27%)	12 (16.67%)	0.728
AKI, *n* (%)	51 (68.92%)	21 (29.17%)	<0.001
DIC, *n* (%)	3 (4.05%)	5 (6.94%)	0.490
Septic shock, *n* (%)	50 (67.57%)	29 (40.28%)	0.001
ACS/MI, *n* (%)	1 (1.35%)	7 (9.72%)	0.032
Mortality, *n* (%)	62 (83.78%)	18 (25.00%)	<0.001
ICU length of stay, days	31 (17–50)	44 (23–57.5)	0.012
Days from start of active therapy to first negative culture, days	4 (2.25–7)	4 (2–7)	0.643

Abbreviations: ICU, intensive care unit; ARDS, acute respiratory distress syndrome; AKI, acute kidney injury; DIC, disseminated intravascular coagulation; ACS, acute coronary syndrome; MI, myocardial infarction.

**Table 5:** Multivariable Fine–Gray competing-risks regression for ICU death (competing event: discharge alive).

Covariate	sHR	95% CI	*p*-Value
Era (Post-COVID vs. Pre-COVID)	0.20	0.11–0.35	<0.001
Age (per 1-year increase)	1.00	0.98–1.02	0.91
CCI (per 1-point increase)	1.09	0.96–1.24	0.16
SOFA score (per 1-point increase)	1.12	1.04–1.20	0.0015

Abbreviations: sHR, subdistribution hazard ratio; CI, confidence interval; CCI, Charlson Comorbidity Index; SOFA, Sequential Organ Failure Assessment.

**Table 6:** Univariate and multivariable analysis of factors associated with 28-day ICU mortality from MDR BSI onset.

Variable	Unadjusted				Adjusted	
	Odds Ratio	95% Confidence Interval	*p*-Value	Odds Ratio	95% Confidence Interval	*p*-Value
Era (Post vs. Pre)	0.08	0.03–0.18	<0.001	0.11	0.04–0.28	**<0.001**
Age	1.03	1.01–1.05	0.009	0.98	0.95–1.02	0.284
CCI	1.32	1.15–1.54	<0.001	1.31	1.04–1.69	**0.027**
SOFA score	1.27	1.14–1.42	<0.001	1.22	1.07–1.40	**0.003**

Abbreviations: ICU, intensive care unit; MDR, multidrug-resistant; CCI, Charlson Comorbidity Index; SOFA, Sequential Organ Failure Assessment; OR, odds ratio; CI, confidence interval.

## Data Availability

The data generated and analyzed during the current study were retrieved from the hospitals’ archives and the electronic patient record system for laboratory results. Due to patient privacy and ethical considerations, these data are not publicly available. Access may be granted upon request to the corresponding author, which is subject to approval by the relevant institutional review boards.

## Supplementary Materials

The following supporting information can be downloaded at: https://www.mdpi.com/article/10.3390/microorganisms14092098/s1, Table S1. Second and third bacteremia related pathogens.

## Abbreviations

The following abbreviations are used in this manuscript:

Abbreviation	Full Term
*A. baumannii*	*Acinetobacter baumannii*
ACS	Acute Coronary Syndrome
AKI	Acute Kidney Injury
ALT	Alanine Aminotransferase
AMR	Antimicrobial Resistance
APACHE II	Acute Physiology and Chronic Health Evaluation II
ARDS	Acute Respiratory Distress Syndrome
AST	Aspartate Aminotransferase
BMI	Body Mass Index
BSI	Bloodstream Infection
*C. auris*	*Candida auris*
*C. parapsilosis*	*Candida parapsilosis*
CCI	Charlson Comorbidity Index
CDC	Centers for Disease Control and Prevention
CI	Confidence Interval
CIF	Cumulative Incidence Function
CLABSI	Central Line-Associated Bloodstream Infection
CNS	Central Nervous System
COVID-19	Coronavirus Disease 2019
CPE	Carbapenemase-Producing Enterobacterales
CRAB	Carbapenem-Resistant *Acinetobacter baumannii*
CRBSI	Catheter-Related Bloodstream Infection
CRP	C-Reactive Protein
CRE	Carbapenem-Resistant Enterobacterales
CRO	Carbapenem-Resistant Organisms
CVC	Central Venous Catheter
DIC	Disseminated Intravascular Coagulation
ECMO	Extracorporeal membrane oxygenation
*E. faecium*	*Enterococcus faecium*
ESBL	Extended-Spectrum β-Lactamase
EUCAST	European Committee on Antimicrobial Susceptibility Testing
ESCMID	European Society of Clinical Microbiology and Infectious Diseases
GI	Gastrointestinal
HABSI	Hospital-Acquired Bloodstream Infection
HAI	Healthcare-Associated Infection
HR	Hazard Ratio
ICU	Intensive Care Unit
IDSA	Infectious Diseases Society of America
INR	International Normalized Ratio
IPC	Infection Prevention and Control
IQR	Interquartile Range
IRB	Institutional Review Board
*K. pneumoniae*	*Klebsiella pneumoniae*
KPC	*Klebsiella pneumoniae* Carbapenemase
MDR	Multidrug-Resistant
MIC	Minimum Inhibitory Concentration
MI	Myocardial Infarction
MR-CNS	Methicillin-Resistant Coagulase-Negative Staphylococci
MRSA	Methicillin-Resistant *Staphylococcus aureus*
NDM	New Delhi Metallo-β-Lactamase
NE	Not Estimable
OR	Odds Ratio
*P. aeruginosa*	*Pseudomonas aeruginosa*
PDR	Pandrug-Resistant
PICC	Peripherally Inserted Central Catheter
RTI	Respiratory Tract Infection
SARS-CoV-2	Severe Acute Respiratory Syndrome Coronavirus 2
sHR	Subdistribution Hazard Ratio
SIRS	Systemic Inflammatory Response Syndrome
SOFA	Sequential Organ Failure Assessment
UTI	Urinary Tract Infection
VRE	Vancomycin-Resistant *Enterococcus*
WBC	White Blood Cell
WHONET	World Health Organization Network for Surveillance of Antimicrobial Resistance
XDR	Extensively Drug-Resistant

## References

[1] Naghavi M., Vollset S.E., Ikuta K.S., Swetschinski L.R., Gray A.P., Wool E.E., Robles Aguilar G., Mestrovic T., Smith G., Han C. (2024). Global burden of bacterial antimicrobial resistance 1990–2021: A systematic analysis with forecasts to 2050. Lancet.

[2] Barišić V., Kovačević T., Travar M., Golić Jelić A., Kovačević P., Vučićević K., Milaković D., Škrbić R. (2025). The Impact of the COVID-19 Pandemic on Antimicrobial Resistance Trends in a Tertiary Care Teaching Hospital: A Ten-Year Surveillance Study. Antibiotics.

[3] Neri J.E.C., Monteiro J., Rodrigues L.L.M., Sobrinho E.B., Monte C.G., Boulhosa A.C.P., Ferreira R.C.A., Sousa R.C.M. (2026). Antimicrobial resistance before and after the COVID-19 pandemic in Northern Brazil. Antimicrob. Steward. Healthc. Epidemiol..

[4] Micheli G., Sangiorgi F., Catania F., Chiuchiarelli M., Frondizi F., Taddei E., Murri R. (2023). The Hidden Cost of COVID-19: Focus on Antimicrobial Resistance in Bloodstream Infections. Microorganisms.

[5] Ntziora F., Giannitsioti E. (2024). Bloodstream infections in the era of the COVID-19 pandemic: Changing epidemiology of antimicrobial resistance in the intensive care unit. J. Intensive Med..

[6] Mateescu D.M., Ilie A.C., Cotet I., Guse C., Muresan C.O., Pah A.M., Badalica-Petrescu M., Iurciuc S., Craciun M.L., Avram A. (2025). Global Burden of Bloodstream Infections in COVID-19: Prevalence, Antimicrobial Resistance, and Mortality Risk. Viruses.

[7] Liu Y., Song H., Wu Y., Liu L., Li N., Zhang M., Li Y., Meng X. (2025). Influence of COVID-19 pandemic on the distribution and drug resistance of pathogens in patients with bloodstream infection. Front. Public Health.

[8] Sakagianni A., Koufopoulou C., Koufopoulos P., Feretzakis G., Koumaki V. (2025). The Impact of COVID-19 on the Epidemiology of Carbapenem Resistance. Antibiotics.

[9] Centers for Disease Control and Prevention (2024). Antimicrobial Resistance Threats in the United States, 2021–2022. https://www.cdc.gov/antimicrobial-resistance/data-research/threats/update-2022.html.

[10] Petrakis V., Panopoulou M., Rafailidis P., Lemonakis N., Lazaridis G., Terzi I., Papazoglou D., Panagopoulos P. (2023). The Impact of the COVID-19 Pandemic on Antimicrobial Resistance and Management of Bloodstream Infections. Pathogens.

[11] Palaiopanos K., Krystallaki D., Mellou K., Kotoulas P., Kavakioti C.A., Vorre S., Vertsioti G., Gkova M., Maragkos A., Tryfinopoulou K. (2024). Healthcare-associated infections and antimicrobial use in acute care hospitals in Greece, 2022; results of the third point prevalence survey. Antimicrob. Resist. Infect. Control.

[12] Eryılmaz Eren E., Iosifidis E., Sav H., Celik I., Mouloudi E., Pyrpasopoulou A., Zarras C., Roilides E. (2026). Candidemia Epidemiology and Antifungal Resistance in Critically-ill Patients During and After COVID-19 Pandemic: Comparison Between Two Hospitals in Greece and Türkiye. Mycopathologia.

[13] Ioannou P., Astrinaki E., Vitsaxaki E., Bolikas E., Christofaki D., Salvaraki A., Lagoudaki E., Ioannidou E., Karakonstantis S., Saplamidou S. (2022). A Point Prevalence Survey of Healthcare-Associated Infections and Antimicrobial Use in Public Acute Care Hospitals in Crete, Greece. Antibiotics.

[14] Koumaki V., Voudanta E., Michelaki A., Orfanidou M., Vagiakou E., Vrioni G., Tsakris A. (2025). Epidemiological Trends of Carbapenemase-Producing *Pseudomonas aeruginosa* in a Tertiary Care Hospital in Athens, Greece, During 2020–2023. Antibiotics.

[15] Mylona E., Kostourou S., Giankoula D., Kolokotroni C., Tsilikis P., Koudoumnakis N., Papagianni M., Kounatidis D., Vallianou N., Perivolioti E. (2026). A Seven-Year Study of Carbapenem-Resistant *Klebsiella pneumoniae* Bloodstream Infections in a Tertiary Hospital in Greece: A Shift Toward Metallo-β-Lactamase and Dual Carbapenemase Strains. Antibiotics.

[16] Keskin Sarıtaş Ç., Özsüt H., Benli A., Başaran S. (2025). Differences between COVID-19 and non-COVID-19 patients’ bloodstream infections: A single-center retrospective study. J. Infect. Dev. Ctries..

[17] Piantoni A., Houard M., Piga G., Zebian G., Ruffier des Aimes S., Holik B., Wallet F., Rouzé A., Kreitmann L., Loiez C. (2023). Relationship between COVID-19 and ICU-Acquired Bloodstream Infections Related to Multidrug-Resistant Bacteria. Antibiotics.

[18] Giacobbe D.R., Battaglini D., Ball L., Brunetti I., Bruzzone B., Codda G., Crea F., De Maria A., Dentone C., Di Biagio A. (2020). Bloodstream infections in critically ill patients with COVID-19. Eur. J. Clin. Invest..

[19] Bongiovanni M., Barda B. (2023). *Pseudomonas aeruginosa* Bloodstream Infections in SARS-CoV-2 Infected Patients: A Systematic Review. J. Clin. Med..

[20] Wilson Dib R., Spallone A., Khawaja F., Feldman A., Cantu S., Chemaly R.F. (2023). The impact of the COVID-19 pandemic on hospital-acquired infections at a comprehensive cancer center. Am. J. Infect. Control.

[21] Peake S.L., Freund Y., Azoulay E., De Pascale G., Gong M.N., Rochwerg B., Hashmi M., Schorr C.A., Mohr N.M., Doi K. (2026). Surviving Sepsis Campaign: International Guidelines for Management of Sepsis and Septic Shock 2026. Crit. Care Med..

[22] Unic-Stojanovic D., Kangrga N., Cirkovic I., Malesevic I., Djokovic Mrdakovic I., Petrovic J., Bojic M. (2026). Sepsis Diagnosis in the Intensive Care Unit: A Comparative Study of Rapid Molecular Diagnostics and Conventional Blood Cultures. Biomedicines.

[23] Wang Y., Lindsley K., Bleak T.C., Jiudice S., Uyei J., Gu Y., Wang Y., Timbrook T.T., Balada-Llasat J.-M. (2025). Performance of molecular tests for diagnosis of bloodstream infections in the clinical setting: A systematic literature review and meta-analysis. Clin. Microbiol. Infect..

[24] Tamma P.D., Heil E.L., Justo J.A., Mathers A.J., Satlin M.J., Bonomo R.A. (2024). Infectious Diseases Society of America 2024 Guidance on the Treatment of Antimicrobial-Resistant Gram-Negative Infections. Clin. Infect. Dis..

[25] Paul M., Carrara E., Retamar P., Tängdén T., Bitterman R., Bonomo R.A., de Waele J., Daikos G.L., Akova M., Harbarth S. (2022). European Society of Clinical Microbiology and Infectious Diseases (ESCMID) guidelines for the treatment of infections caused by multidrug-resistant Gram-negative bacilli (endorsed by European society of intensive care medicine). Clin. Microbiol. Infect..

[26] Tamma P.D., Bonomo R.A., Heil E.L., Justo J.A., Satlin M.J., Mathers A.J. (2026). Infectious Diseases Society of America 2026 Guidance on the Treatment of Antimicrobial-Resistant Gram-Negative Infections. Clin. Infect. Dis..

[27] Papamichalis P., Oikonomou K.G., Xanthoudaki M., Valsamaki A., Skoura A.L., Papathanasiou S.K., Chovas A. (2024). Extracorporeal organ support for critically ill patients: Overcoming the past, achieving the maximum at present, and redefining the future. World J. Crit. Care Med..

[28] Qadir N., Sahetya S., Munshi L., Summers C., Abrams D., Beitler J., Bellani G., Brower R.G., Burry L., Chen J.T. (2024). An Update on Management of Adult Patients with Acute Respiratory Distress Syndrome: An Official American Thoracic Society Clinical Practice Guideline. Am. J. Respir. Crit. Care Med..

[29] Kołodziejczak M.M., Sierakowska K., Tkachenko Y., Kowalski P. (2023). Artificial Intelligence in the Intensive Care Unit: Present and Future in the COVID-19 Era. J. Pers. Med..

[30] Magiorakos A.P., Srinivasan A., Carey R.B., Carmeli Y., Falagas M.E., Giske C.G., Harbarth S., Hindler J.F., Kahlmeter G., Olsson-Liljequist B. (2012). Multidrug-resistant, extensively drug-resistant and pandrug-resistant bacteria: An international expert proposal for interim standard definitions for acquired resistance. Clin. Microbiol. Infect..

[31] Arendrup M.C., Patterson T.F. (2017). Multidrug-Resistant *Candida*: Epidemiology, Molecular Mechanisms, and Treatment. J. Infect. Dis..

[32] Hellenic Society for Infectious Diseases (2025). Recommendations for the Diagnosis and Treatment of Intravascular Catheter-Related Infections. https://www.loimoxeis.gr.

[33] Charlson M.E., Pompei P., Ales K.L., MacKenzie C.R. (1987). A new method of classifying prognostic comorbidity in longitudinal studies: Development and validation. J. Chronic. Dis..

[34] Vincent J.L., Moreno R., Takala J., Willatts S., De Mendonça A., Bruining H., Reinhart C.K., Suter P.M., Thijs L.G. (1996). The SOFA (Sepsis-related Organ Failure Assessment) score to describe organ dysfunction/failure. On behalf of the Working Group on Sepsis-Related Problems of the European Society of Intensive Care Medicine. Intensive Care Med..

[35] Wagner D.P., Draper E.A. (1984). Acute physiology and chronic health evaluation (APACHE II) and Medicare reimbursement. Health Care Financ. Rev..

[36] Al-Hasan M.N., Baddour L.M. (2020). Resilience of the Pitt Bacteremia Score: 3 Decades and Counting. Clin. Infect. Dis..

[37] Baddam S., Burns B. (2026). Systemic Inflammatory Response Syndrome. StatPearls.

[38] The European Committee on Antimicrobial Susceptibility Testing (EUCAST) (2026). Breakpoint Tables for Interpretation of MICs and Zone Diameters, Version 16.0. https://www.eucast.org/bacteria/clinical-breakpoints-and-interpretation/clinical-breakpoint-tables/.

[39] Iglesias R., Badia J.M., Vela E., Yébenes J.C. (2025). Baseline morbidity and chronic medications as determinants of sepsis outcomes: Focus on statins, corticosteroids, and NSAIDs in a population-based cohort of 59,578 patients. Front. Pharmacol..

[40] Luo H., Yang X., Chen K., Lan S., Liao G., Xu J. (2022). Blood creatinine and urea nitrogen at ICU admission and the risk of in-hospital death and 1-year mortality in patients with intracranial hemorrhage. Front. Cardiovasc. Med..

[41] Perel N., Taha L., Farkash R., Steinmetz Y., Shaheen F., Levi N., Dadon Z., Karameh H., Karmi M., Maller T. (2022). Level of Hypoalbuminemia as a Prognostic Factor in Patients admitted to a Tertiary Care Intensive Coronary Care Unit. Cardiol. Cardiovasc. Med..

[42] Wang L.N., He D.K., Shao Y.R., Lv J., Wang P.F., Ge Y., Yan W. (2023). Early platelet level reduction as a prognostic factor in intensive care unit patients with severe aspiration pneumonia. Front. Physiol..

[43] Brinkman S., de Keizer N.F., de Lange D.W., Dongelmans D.A., Termorshuizen F., van Bussel B.C.T. (2024). Strain on Scarce Intensive Care Beds Drives Reduced Patient Volumes, Patient Selection, and Worse Outcome: A National Cohort Study. Crit. Care Med..

[44] Gristina G.R., Piccinni M. (2021). COVID-19 pandemic in ICU. Limited resources for many patients: Approaches and criteria for triaging. Minerva Anestesiol..

[45] Leclerc T., Donat N., Donat A., Pasquier P., Libert N., Schaeffer E., D’Aranda E., Cotte J., Fontaine B., Perrigault P.F. (2020). Prioritisation of ICU treatments for critically ill patients in a COVID-19 pandemic with scarce resources. Anaesth. Crit. Care Pain. Med..

[46] Baltali S., Firat A. (2023). The impact of COVID-19 pandemic on the indications of non-COVID-19 obstetric and gynecological admissions to the intensive care unit (ICU) and its overall consequences. Sci. Rep..

[47] Watanabe S., Shin J.H., Okuno T., Morishita T., Takada D., Kunisawa S., Imanaka Y. (2022). Medium-term impacts of the waves of the COVID-19 epidemic on treatments for non-COVID-19 patients in intensive care units: A retrospective cohort study in Japan. PLoS ONE.

[48] Moin E.E., Seewald N.J., Halpern S.D. (2025). Use of Life Support and Outcomes Among Patients Admitted to Intensive Care Units. JAMA.

[49] Diekema D.J., Hsueh P.R., Mendes R.E., Pfaller M.A., Rolston K.V., Sader H.S., Jones R.N. (2019). The Microbiology of Bloodstream Infection: 20-Year Trends from the SENTRY Antimicrobial Surveillance Program. Antimicrob. Agents Chemother..

[50] Abdelaziz Abdelmoneim S., Mohamed Ghazy R., Anwar Sultan E., Hassaan M.A., Anwar Mahgoub M. (2024). Antimicrobial resistance burden pre and post-COVID-19 pandemic with mapping the multidrug resistance in Egypt: A comparative cross-sectional study. Sci. Rep..

[51] Polemis M., Tryfinopoulou K., Giakkoupi P., Vatopoulos A. (2020). Eight-year trends in the relative isolation frequency and antimicrobial susceptibility among bloodstream isolates from Greek hospitals: Data from the Greek Electronic System for the Surveillance of Antimicrobial Resistance-WHONET-Greece, 2010 to 2017. Euro Surveill..

[52] Kypraiou D., Sourris A., Astrinaki E., Vitsaxaki E., Saplamidou S., Vakonaki M., Tryfinopoulou K., Chamilos G., Ioannou P., Kofteridis D. (2026). Temporal Trends and Mortality of Vancomycin-Resistant *Enterococcus* Bacteremia-A Six-Year Retrospective Cohort Study in a Tertiary Hospital in Greece. Pathogens.

[53] Koumaki V., Voudanta E., Michelaki A., Orfanidou M., Vagiakou E., Vrioni G., Tsakris A. (2025). Changing Epidemiology of Carbapenemases Among Carbapenem-Resistant *Enterobacterales* in a Greek Tertiary Care Hospital in Athens, 2020 to 2023. Antibiotics.

[54] Vincent J.-L., Rello J., Marshall J., Silva E., Anzueto A., Martin C.D., Moreno R., Lipman J., Gomersall C., Sakr Y. (2009). International Study of the Prevalence and Outcomes of Infection in Intensive Care Units. JAMA.

[55] Petit H., de Tymowski C., Dudoignon E., Liberge M., Donay J.-L., Chaussard M., Coutrot M., Cupaciu A., Guillemet L., Deniau B. (2025). Epidemiology and Outcomes of Bloodstream Infections in Patients in a Burns Intensive Care Unit: An 8-Year Retrospective Study. Open Forum Infect. Dis..

[56] Otto M. (2009). *Staphylococcus epidermidis*—the ‘accidental’ pathogen. Nat. Rev. Microbiol..

[57] Gowardman J.R., Robertson I.K., Parkes S., Rickard C.M. (2008). Influence of insertion site on central venous catheter colonization and bloodstream infection rates. Intensive Care Med..

[58] Cosme V., Massart N., Reizine F., Machut A., Vacheron C.H., Savey A., Friggeri A., Lepape A. (2024). Central venous catheter-related infection: Does insertion site still matter? A French multicentric cohort study. Intensive Care Med..

[59] He X., Li C., Wang Z., Yang M., Zhou T., Gu Y., Zhang Y., Wang W., Hu Y. (2026). The risk of central line-associated bloodstream infection between internal jugular and subclavian sites in critically ill patients: A multicenter cohort study. BMC Infect. Dis..

[60] Centers for Disease Control and Prevention 2024 National and State Healthcare-Associated Infections Progress Report. https://www.cdc.gov/healthcare-associated-infections/php/data/progress-report.html.

[61] Conroy O.D., Mazzella A., Choi H., Elmes J., Wilson M., Chudasama D.Y., Gerver S.M., Mihalkova M., Rhodes A., Wilson A.P.R. (2025). Bloodstream Infections in Critical Care Units in England, April 2017 to March 2023: Results from the First Six Years of a National Surveillance Programme. Microorganisms.

[62] European Centre for Disease Prevention and Control Proposal for EU Guidance on the Establishment and Implementation of Infection Prevention and Control Programmes in Healthcare. https://www.ecdc.europa.eu/en/publications-data/proposal-eu-guidance-infection-prevention-and-control.

[63] World Health Organization Global Report on Infection Prevention and Control 2024. https://www.who.int/publications/i/item/9789240103986?utm_source.

[64] Schinas G., Skintzi K., De Lastic A.L., Rodi M., Gogos C., Mouzaki A., Akinosoglou K. (2023). Patterns, Cost, and Immunological Response of MDR vs. Non MDR-Bacteremia: A Prospective Cohort Study. Pathogens.

[65] Lye D.C., Earnest A., Ling M.L., Lee T.E., Yong H.C., Fisher D.A., Krishnan P., Hsu L.Y. (2012). The impact of multidrug resistance in healthcare-associated and nosocomial Gram-negative bacteraemia on mortality and length of stay: Cohort study. Clin. Microbiol. Infect..

[66] Wang W., Jiang T., Zhang W., Li C., Chen J., Xiang D., Cao K., Qi L.W., Li P., Zhu W. (2017). Predictors of mortality in bloodstream infections caused by multidrug-resistant gram-negative bacteria: 4 years of collection. Am. J. Infect. Control.

[67] Kudo D., Kushimoto S., Miyagawa N., Sato T., Hasegawa M., Ito F., Yamanouchi S., Honda H., Andoh K., Furukawa H. (2018). The impact of organ dysfunctions on mortality in patients with severe sepsis: A multicenter prospective observational study. J. Crit. Care.

[68] Holmes C.L., Albin O.R., Mobley H.L.T., Bachman M.A. (2025). Bloodstream infections: Mechanisms of pathogenesis and opportunities for intervention. Nat. Rev. Microbiol..

[69] Harbarth S., Ferrière K., Hugonnet S., Ricou B., Suter P., Pittet D. (2002). Epidemiology and Prognostic Determinants of Bloodstream Infections in Surgical Intensive Care. Arch. Surg..

[70] Zahar J.R., Timsit J.F., Garrouste-Orgeas M., Français A., Vesin A., Descorps-Declere A., Dubois Y., Souweine B., Haouache H., Goldgran-Toledano D. (2011). Outcomes in severe sepsis and patients with septic shock: Pathogen species and infection sites are not associated with mortality. Crit. Care Med..

[71] Kumar A., Kumar A., Tyagi A., Singh R., Charaya M.U. (2026). A review of bloodstream infections−pathogens, pathogenesis, diagnostic strategies, treatment methods−challenges and future aspects. Eur. J. Clin. Microbiol. Infect. Dis..

[72] Vonineng N., Sutherasan Y., Bruminhent J. (2025). Clinical characteristics, risk factors and outcome of critically ill immunocompromised patients with bloodstream infections and sepsis. PLoS ONE.

[73] Nagendra D., Eshwara V.K., Chaudhuri S., Shanbhag V., Srinivas T. (2025). Predictors of mortality in sepsis patients with *Acinetobacter baumannii* and *Klebsiella pneumoniae* bacteremia. Clin. Epidemiol. Glob. Health.

[74] Haldane V., De Foo C., Abdalla S.M., Jung A.-S., Tan M., Wu S., Chua A., Verma M., Shrestha P., Singh S. (2021). Health systems resilience in managing the COVID-19 pandemic: Lessons from 28 countries. Nat. Med..

[75] Ravaghi H., Khalil M., Al-Badri J., Naidoo A.V., Ardalan A., Khankeh H. (2022). Role of hospitals in recovery from COVID-19: Reflections from hospital managers and frontliners in the Eastern Mediterranean Region on strengthening hospital resilience. Front. Public Health.

[76] Behrens D.A., Rauner M.S., Sommersguter-Reichmann M. (2022). Why Resilience in Health Care Systems is More than Coping with Disasters: Implications for Health Care Policy. Schmalenbach Z. Betriebswirtsch Forsch..

[77] Goniewicz K., Khorram-Manesh A. (2026). Transformative Resilience in European Health Governance After COVID-19: A Policy Analysis. Healthcare.

