# Supplementary material for: Multidrug-Resistant Bloodstream Infections Before and After COVID-19: A Temporal Assessment of Outcomes and Resistance Across Two Eras in a Non-COVID Intensive Care Unit—The MATURATE ICU Study
Source: Microorganisms. 2026 Sep 19;14(9):2098. doi: 10.3390/microorganisms14092098 (PMC13609234; doi:10.3390/microorganisms14092098)
Supplement: Supplementary file 1 [file microorganisms-14-02098-s001.zip › Table S1.pdf]

Table S1. Second and third bacteremia related pathogens.

	Era		
	**Pre COVID-19 (2017–2020), n=74**	**Post COVID-19 (2022–2025), n=72**	**p**
Second pathogen			0.071
S. epidermidis, n (%)	11 (14.9%)	10 (13.9%)	
P. aeruginosa, n (%)	6 (8.1%)	5 (6.9%)	
A. baumannii, n (%)	4 (5.4%)	3 (4.2%)	
C. parapsilosis, n (%)	4 (5.4%)	3 (4.2%)	
S. haemolyticus, n (%)	3 (4.1%)	3 (4.2%)	
K. pneumoniae, n (%)	4 (5.4%)	1 (1.4%)	
C. auris, n (%)	0 (0.0%)	4 (5.6%)	
E. faecium, n (%)	1 (1.4%)	3 (4.2%)	
S. capitis, n (%)	3 (4.1%)	1 (1.4%)	
Others, n (%)	4 (5.4%)	16 (22.2%)	
Third pathogen			0.315
C. parapsilosis, n (%)	4 (5.4%)	4 (5.6%)	
A. baumannii, n (%)	5 (6.8%)	1 (1.4%)	
S. epidermidis, n (%)	4 (5.4%)	2 (2.8%)	
P. aeruginosa, n (%)	2 (2.7%)	1 (1.4%)	
S. capitis, n (%)	1 (1.4%)	2 (2.8%)	
S. haemolyticus, n (%)	0 (0.0%)	3 (4.2%)	
Others, n (%)	4 (5.4%)	8 (11.1%)	

Abbreviations: *S. epidermidis, Staphylococcus epidermidis; P. aeruginosa, Pseudomonas aeruginosa; A. baumannii, Acinetobacter baumannii; C. parapsilosis, Candida parapsilosis; S. haemolyticus, Staphylococcus haemolyticus; K. pneumoniae, Klebsiella pneumoniae; C. auris, Candida auris; E. faecium, Enterococcus faecium; S. capitis, Staphylococcus capitis*.
